# Acute management of massive pulmonary embolism in pregnancy

**DOI:** 10.3389/fgwh.2024.1473405

**Published:** 2025-01-06

**Authors:** Shahin Qadri, Ashwini Bilagi, Abha Sinha, Derek Connolly, Richard Murrin, Shagaf Bakour

**Affiliations:** ^1^ Department of Obstetrics and Gynecology, Sandwell and West Birmingham NHS Trust, Birmingham, United Kingdom; ^2^ Aston Medical School, Aston University, Birmingham, United Kingdom; ^3^ Birmingham Medical School, University of Birmingham, Birmingham, United Kingdom; ^4^ Department of Cardiology, Sandwell and West Birmingham NHS Trust, Birmingham, United Kingdom; ^5^ Department of Hematology, Sandwell and West Birmingham NHS Trust, Birmingham, United Kingdom

**Keywords:** pregnancy, pulmonary embolism, thrombolysis, ECMO, embolectomy

## Abstract

**Key content:**

• Massive pulmonary embolism (PE) during pregnancy or the postpartum period is a rare but potentially lethal event.
• Physiological changes in the coagulation system during pregnancy and puerperium would lead to a hypercoagulable state.
• Diagnosis of PE in pregnancy remains a challenge due to physiological changes in pregnancy. There are no validated scoring systems for assessing pregnant/postpartum women with suspected PE. Massive PE should be suspected in all cases with haemodynamic instability in pregnancy.
• The Management of massive pulmonary embolism should be timely and aggressive. Thrombolysis for massive PE during pregnancy and the postpartum period has shown to be associated with high maternal and fetal survival (94% and 88%). But other therapeutic options such as (catheter [or surgical] thrombectomy, ECMO) should be considered in the postpartum period, given the high risk of major bleeding with thrombolysis.
• Thrombolysis remains the most-used and reasonably successful modality of treatment in pregnancy but should be avoided in the postpartum period as it can cause life-threatening haemorrhage. During the post-partum period, thrombectomy is the treatment of choice.

**Learning objectives:**

• To understand the pathophysiology of massive PE.
• To appreciate the treatment options in pregnancy and postpartum period and their pros and cons.
• To understand the need for further work in this area especially in creating a validated algorithm for diagnosing PE in pregnancy and postpartum period.

## Introduction

About 1 in 1,000–3,000 pregnancies are complicated by pulmonary embolism (PE) (1). Hemodynamically unstable PE as a result of cardiac obstructive shock, its most severe manifestation, may be found in 5% of PEs and is among the leading causes of maternal death in industrialized countries (Sultan et al., 2013, Simcox et al., 2015).

The venous thromboembolism (VTE) clinically presenting as deep vein thrombosis (DVT) or pulmonary embolism (PE), is globally the third most frequent acute cardiovascular syndrome after myocardial infarction and stroke (1). The risk of antenatal VTE is four- to five-fold higher in pregnant women than in nonpregnant women of the same age and VTE remains one of the main direct causes of maternal death in the UK (1). Pulmonary embolism (PE) is a common and potentially deadly form of venous thromboembolic disease. About 1 in 1,000–3,000 pregnancies are complicated by PE (2). VTE in pregnancy remains a leading cause of direct maternal mortality in the developed world and identifiable risk factors are increasing in incidence. VTE is approximately 10-times more common in the pregnant population (compared with non-pregnant women) with an incidence of 1 in 1,000 and the highest risk in the postnatal period.

**Figure 1:**
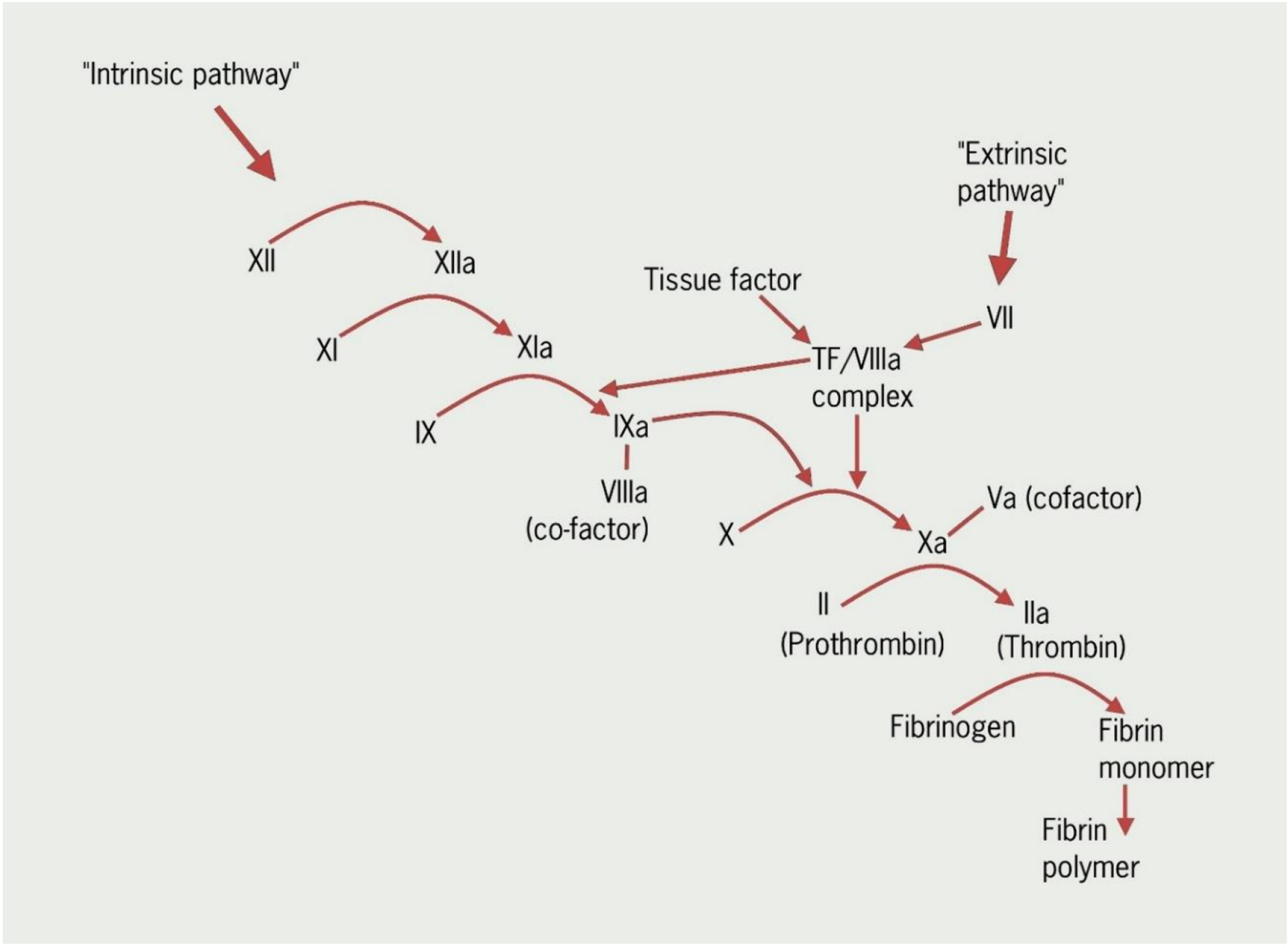
A simplified diagram showing some of the main steps in the coagulation cascade (Courtesy- British Journal of Cardiology, REVISED anticoagulation module 1: the haemostatic system in health and disease).

**Figure 2:**
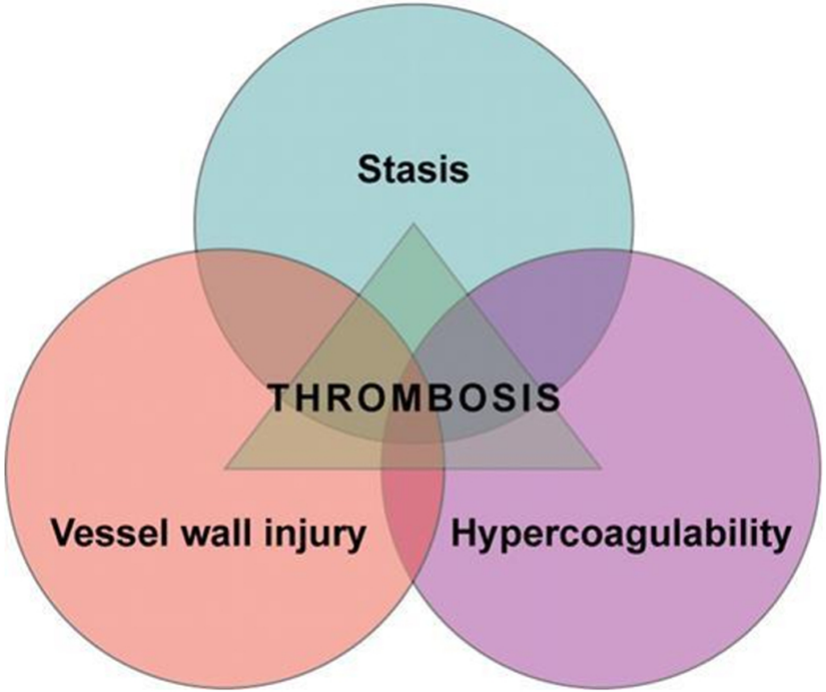
Virchow′s Triad (Courtesy: https://ashpublications.org/blood/article/114/6/1138/26154/is-Virchow-s-triad-complete).

This increased risk reflects the hypercoagulable state of pregnancy that starts with conception and remains high until around 8 weeks postpartum. There is increased venous stasis in the pelvic and lower limb veins due to the vasodilatory effects of pregnancy hormones and physical obstruction from the gravid uterus (3). The three well known components of “Virchow's triad” of venous stasis, hypercoagulability and vascular damage are increased during pregnancy and delivery (Figures 1 and 2).

Recent EMBRACE report suggests that in about two thirds of women who lost their lives during pregnancy postpartum period, it was noted that improvements in care could have made a difference to their outcome. The inconsistency is due to the confusion over interpretation of national guidance and a lack of reassessment at points when women's risk changed during pregnancy (4). The time has come to address this.

### Definition and classification of pulmonary embolism

Pulmonary embolism (PE) is an acute and potentially fatal condition in which an embolic material, usually a thrombus originating from one of the deep veins of the legs or pelvis, blocks one or more pulmonary arteries causing impaired blood flow and increased pressure to the right cardiac ventricle.

Pulmonary embolism can be classified depending on degree of pulmonary vasculature obstructed by the obstruction caused by the blood clots. While classifying pulmonary embolism, it is reasonable to consider not only size of the embolus but also the underlying cardiopulmonary reserve. Therefore, the best way to classify pulmonary embolism depends on the hemodynamic consequences.

Severe PE could be divided in to massive or sub massive PE. Massive pulmonary embolism (PE) is characterized by systemic hypotension (defined as a systolic arterial pressure < 90 mm Hg or a drop in systolic arterial pressure of at least 40 mm Hg for at least 15 min which is not caused by new onset arrhythmias) or shock (manifested by evidence of tissue hypoperfusion and hypoxia, including an altered level of consciousness, oliguria, or cool, clammy extremities) (5). PE is termed as 'sub massive' when associated with right ventricular dilation without reported hemodynamic compromise.

European society of cardiology has termed this group of PEs with haemodynamic instability as “high risk” PE. PE with haemodynamic stability is termed as “low risk” PE (6).

The European society of cardiology has recently produced a multifactorial classification (6).

### Pathophysiology of severe PE

#### Hemodynamics

Majority of the PEs originate as thrombi in the deep veins of the lower extremities. The site of thrombosis is mostly in the calf veins, then femoropopliteal veins, and less frequently in the iliac veins (7). Pelvic vein DVTs can cause PE during pregnancy due to the venous stasis caused by the gravid uterus. Emboli detach from their point of origin and travel through the systemic venous system, through the right sided chambers of the heart, and lodge in the pulmonary arterial system. PE contributes to gas exchange abnormalities and hypoxemia, but it is predominantly the hemodynamic consequences of PE that are responsible for increased morbidity and mortality (8).

Hemodynamic instability and shock are the most important factors leading to death in massive PE.

The hemodynamic response to acute occlusion of the pulmonary vessels depends on several factors that increase pulmonary vascular resistance (PVR) and pressure overload, including clot size and the degree to which clot is centrally positioned. Hypoxia increases PVR and pulmonary artery (PA) pressures along with serotonin, platelet-activating factor, thrombin, vasoactive peptides (C3, C5a), and histamine (9, 10). The increase in PVR translates into an elevated PA pressure as long as cardiac output is sustained.

Raised PVR translates to right ventricular dilatation. The Frank Sterling mechanism would alter the contractile properties of the right ventricle (RV) myocardium, thus maintaining the cardiac output by preserving stroke volume, even if the ejection fraction falls. The contraction time of the RV is prolonged due to the increase in wall tension and myocyte stretch. This causes prolonged contraction time of the RV. This, along with systemic vasoconstriction described above would increase pulmonary artery pressure. This improves the flow through the obstructed pulmonary vascular bed-thus maintaining the blood pressure for some time (6).

However, the extent of this early adaptation is limited. Prolongation of RV contraction time leads to desynchronization of the ventricles which may be exacerbated by the development of right bundle branch block (11). Increased RV size also increases wall stress and tension. Wall stress reduces RV oxygen uptake and, combined with increased oxygen demand, sets the stage for ischemia. The RV dilation, pressure overload, and ischemia together result in a reduced stroke volume and cardiac output. Right ventricle perfusion depends on the pressure gradient between the mean arterial pressure and subendocardial pressure. This, along with increase oxygen demand result in RV ischemia and infarction. Vasoconstrictors may help prevent ischemia for some time by increasing the mean arterial pressure (12). As the RV fails, reduced cardiac output may reduce PA pressures if PVR remains fixed.

#### Impaired gas exchange

Respiratory failure in PE is predominantly a result of the cardiovascular changes described above (13). There is ventilation perfusion mismatch occurs due to the areas of reduced flow in obstructed pulmonary arteries and the areas of overflow in the capillary bed served by non-obstructed pulmonary vessels. This exacerbates the ongoing going hypoxaemia.

In about one-third of patients, an inverted pressure gradient between the RA and left atrium may lead to severe hypoxaemia, and an increased risk of paradoxical embolization and stroke right-to-left shunting through a patent foramen ovale (13).

### Diagnosis

#### Clinical symptoms and signs

The clinical signs and symptoms of acute PE are non-specific. Diagnosis of PE during pregnancy can be challenging as symptoms frequently overlap with symptoms of normal pregnancy. PE is considered as a possibility in a patient with any or all of the following symptoms -dyspnoea, chest pain, pre-syncope or syncope, or haemoptysis (14).

Massive PE often presents with circulatory collapse, mental status changes, syncope, arrhythmias, seizures, and death (12). Massive PE should be considered as a differential diagnosis in any hypotensive patient with raised central venous pressure, along with other possibilities such as acute myocardial infarction, tension pneumothorax, pericardial tamponade, and new onset arrhythmia (5).

Syncope may occur, and is associated with a higher prevalence of haemodynamic instability and RV dysfunction. Dyspnoea may be acute and severe in central PE.

In addition to symptoms, knowledge of the predisposing factors for VTE is important in determining the clinical probability of the disease, which increases with the number of predisposing factors present.

### Risk assessment and investigations

In non-pregnant patients, the combination of clinical features and predisposing risk factors have been incorporated in to the well-established prediction algorithms using pre-test clinical probability assessment tools such as Well's score, Geneva score and pulmonary embolism severity index (15–17). These are backed up with a non-invasive and cost-effective blood test in the form of D-Dimer. Together they rule out up to 30% of patients with suspected PE (18).

**Figure 3:**
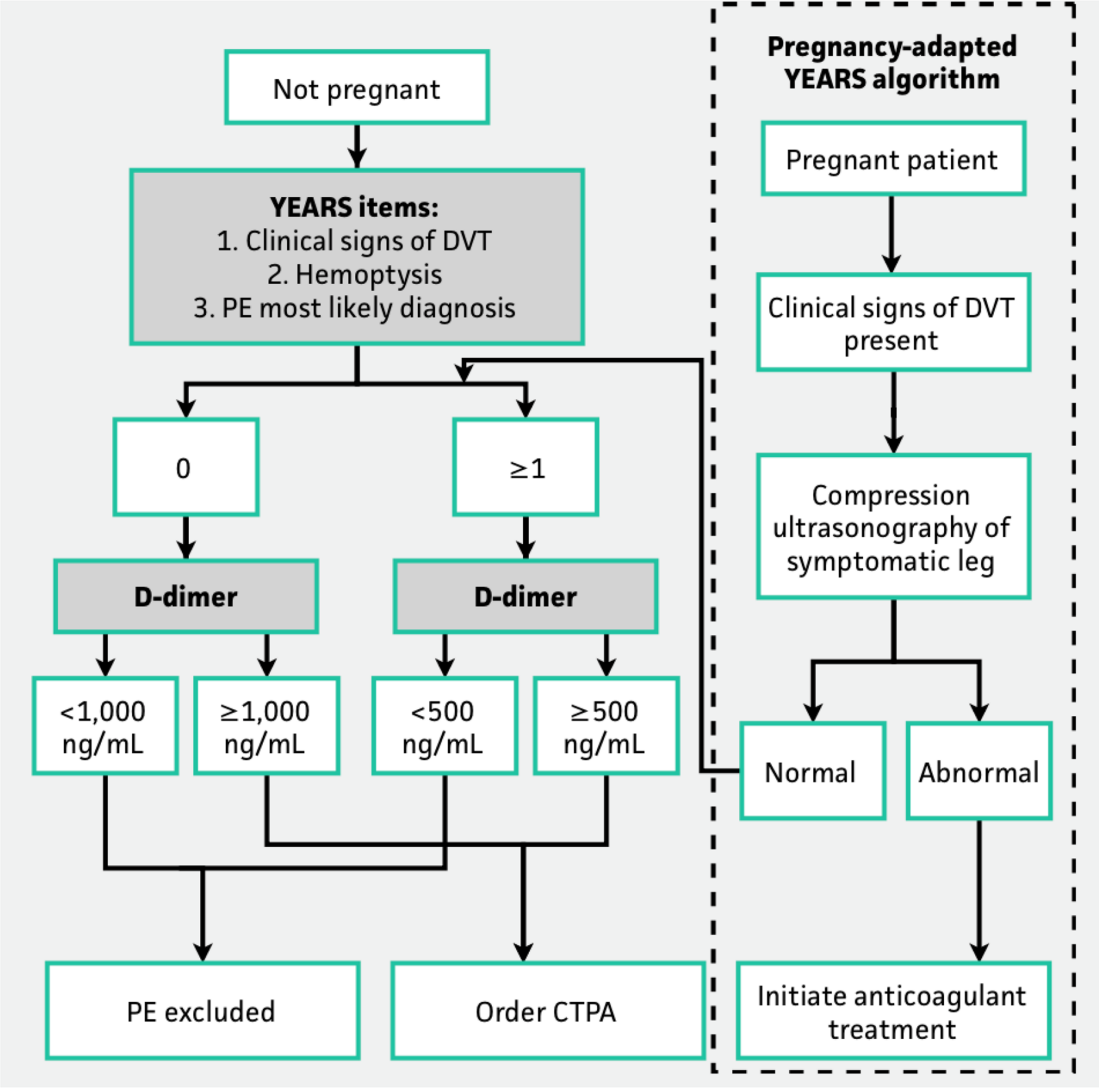
Showing the recent prospective studies on adapted D-dimer levels and YEARS algorithm.

In pregnancy, there are no validated pre-test probability assessment tools to manage PE (19). The D-dimer levels are known to increase in pregnancy and thus have limited value in the diagnostic algorithm if this rise is not accounted for. Recently, there has been some efforts to adopt this in the algorithm by creating different cut-offs of D-Dimer levels in various stages of pregnancy but his is yet to be validated (Figure 3). There has been some recent interest in this area (20). A prospective study involving 498 women looked in to a pregnancy- adapted YEARS algorithm with D-dimer levels. PE was ruled out without CTPA in women deemed to be at low PE risk according to the combination of the algorithm and D-dimer results (what D-dimer threshold quoted by this study to exclude PE in low riskers). At 3 months, only one woman with PE excluded on the basis of the algorithm developed a popliteal DVT and no women developed PE (20). Another recent multinational prospective study involving 441 pregnancies has come up with a diagnostic strategy–based on the assessment of clinical probability, D-dimer measurement, CUS, and CTPA–may safely exclude PE in pregnancy. In this study, it was possible to exclude PE based on of a negative D-dimer result without imaging in 11.7% of the 392 women with a non-high pre-test probability (Geneva) score, a rate that was reduced to 4.2% in the third trimester.

Recently the ESC has proposed a diagnostic algorithm including D-Dimer to diagnose PE in pregnancy. But that appears to be more relevant to the PE wherein haemodynamic stability is maintained (6).

### Imaging tests

Recent ESC recommends in suspected high-risk PE, as indicated by the presence of haemodynamic instability, bedside echocardiography or emergency CTPA (depending on availability and clinical circumstances) is recommended for diagnosis (1).

### Treatment approaches

In women with acute pulmonary embolism who are haemodynamically stable, the conventional treatment of choice is low molecular weight heparin (LMWH). The other possible treatment option is unfractionated heparin (UFH). LMWH has more predictable pharmacokinetics compared to UFH and a more favourable risk profile. Vitamin K Antagonists (VKA) and non-vitamin K antagonist oral anticoagulants (NOAC) cross the placenta, and consequently confer a risk of fetal haemorrhage or teratogenicity and hence are not advocated as treatment options (6).

However severe PE warrants more aggressive approach to remove the clot to aid survival. Early diagnosis is vital. The interval from the onset of symptoms to death is incredibly short. In patients with massive PE, 50% die within 30 min, 70% die within 1 h, and more than 85% die within 6 h of the onset of symptoms (21).

Therapies used in massive PE to rapidly reverse PA obstruction include thrombolysis, catheter-directed therapies, and surgical embolectomy. These definitive therapies offer rapid reversal of PA occlusion, should reduce PVR and reduce RV pressure overload; thus restoring normal hemodynamics (22). Extracorporeal membrane oxygenation ECMO is another potential option (6).

(1) Thrombolysis

Thrombolysis is a process of administering thrombolytic agents either administered systemically or to a specific target site via catheters. Thrombolytic agents act by converting plasminogen to plasmin, an active enzyme. Broadly there are two types of thrombolytic agents- Nonspecific plasminogen activators and fibrin specific plasminogen activators. The non-specific plasminogen activators (example urokinase, streptokinase) do not discriminate between fibrin-bound and circulating plasminogen and can trigger a systemic lytic state whereas the fibrin-specific plasminogen activators (e.g., tissue plasminogen activator, alteplase, reteplase preferentially activate fibrin bound plasminogen.

This is a well-established treatment for PE in pregnancy. Thrombolytic therapy leads to faster improvements in pulmonary obstruction, Pulmonary artery pressure, and PVR in patients with PE and these improvements are accompanied by a reduction in RV dilation on echocardiography [276–279]. The major risks of using thrombolytic therapy are life-threatening haemorrhage and distal embolization. The ideal thrombolytic agent would induce local pathological clot dissolution without producing systemic effects. The most commonly used thrombolytic agent in pregnancy described in the literature is alteplase (23). Both the systematic and the catheter directed administered therapy have been reported (24).

There is well established evidence including randomized controlled trials in non-pregnant patients that thrombolysis is a well-stablished treatment for PE (25–28). The greatest benefit is observed when treatment is initiated within 48 h of symptom onset, but thrombolysis can still be useful in patients who have had symptoms for 6–14 days (25). As the Thrombolytic agents are considerably big molecules weighing more than 1,000 Daltons, they are unlikely to cross the placenta (29). A recent meta-analysis reported 83 women treated with thrombolysis for PE in the pregnant and post-partum period. The survival rate was 94%. The risk of major bleeding was 17.5% during pregnancy and 58.3% in the postpartum period, mainly because of severe postpartum haemorrhages. Fetal deaths possibly related to PE or its treatment occurred in 12.0% of cases treated during pregnancy (23).

#### Challenges in pregnancy

Thrombolysis is relatively contraindicated in pregnancy because of presumed risk of maternal bleeding and risk of fetal loss (30). The recent systematic review examined 83 women with thrombolysis. The risk of major bleeding was 17.5% during pregnancy and 58.3% in the postpartum period, mainly because of severe postpartum haemorrhages. Another review also reports increased major bleeding in post-partum period compared to antepartum period (31). Fetal deaths possibly related to PE or its treatment occurred in 12.0% of cases treated during pregnancy. They reported that more than half of the women had to face major bleeding while only 18 percent of pregnant women suffered major bleeding. Another review agrees with the above findings (32). Because of risks of bleeding, thrombolysis is contraindicated in postpartum period (6). However, a recent RCOG document recommends that thrombolysis should not be regarded as absolutely contraindicated in the post-partum period (33).

(2) Thrombectomy

There are two ways of achieving thrombectomy—Surgical Thrombectomy and Percutaneous catheter thrombectomy. Surgical thrombectomy involves sternotomy, a right atriotomy and then a longitudinal incision into the main pulmonary artery trunk distal to the pulmonary valve with possible further extension of the incision (34). In percutaneous catheter thrombectomy, the access is gained via peripheral vessels such as femoral vessel and the clot is reached endovascularly. The clot can then be mechanically removed or fragmented (fragmentation or thrombo aspiration without thrombolysis). Thrombectomy is another reasonable alternative to treat life threatening PE. The major advantage of this mode of treatment is in postpartum period when there is significant risk of major bleeding (6). Martillotti et al. in 2017, published their meta-analysis with data of 36 women treated by embolectomy. Among these 36 women with surgical thrombectomy, maternal survival and risk of major bleeding were around 86% and 20%, with fetal deaths possibly related to surgery in 20.0% (23). Thus, this modality is the treatment of choice post-partum period and a feasible alternative in the antenatal phase (6).

(3) Extracorporeal membrane oxygenation (ECMO).

ECMO is a life support technique that involves withdrawing venous blood via an active pump and returning oxygenated blood into a major central artery. It functions as a parallel circuit to the patient's heart and lung. The first of such machine was documented to be used in 1951 by Dennis during an open heart operation but unfortunately the patient didn't survive (35). The first successful use of ECMO during an open heart operation is documented in 1953 by Gibbon (36). ECMO can play an important role for patients in cardiogenic shock from massive PE. It serves as a bridge, allowing the right ventricle to recover while providing time for other definitive advanced therapies (e.g., thrombolysis or thrombo-embolectomy) to reduce thrombus burden (37).

There is very limited evidence of its use in pregnancy in the treatment of massive PE.

The recent review reports three cases of massive PE during pregnancy or the postpartum period treated with ECMO and anticoagulation, without thrombolysis or thrombectomy. All the women survived, without major bleeding. In the two antenatal cases, there was one premature delivery but no other reported fetal complications. ECMO was also used as an adjuvant therapy for 3–7 days after thrombolysis in eight women with massive PE (23).

Pregnant women are at high thrombotic risk due to physiological changes during the gestational period. As a consequence, pulmonary embolism can lead to severe respiratory failure associated with or without cardiovascular dysfunction. Again, ECMO is the last option where other treatments fail and will ensure both cardiopulmonary support and adequate anticoagulant therapy (38).

Sharma et al. (2015) published a review on the use of ECMO in pregnant women and in the postpartum period. The authors examined clinical cases of patients with respiratory distress who subsequently developed cardiorespiratory failure requiring ECMO from 2009 to 2014. In that observational study, overall maternal survival was observed to be 80% and fetal survival was 70% (39).

Palella et in 2023 conducted a systematic review in 306 women, 203 were prepartum at the time of cannulation (66.3%), and 103 were postpartum (33.7%). The result obtained suggested that VV-ECMO in this population could save five out of six mothers (survival > 80%), while fetal mortality was doubled with approximately one-third unfavourable outcomes (fetal survival ca. 67.9%) (40).

The literature highlight that the application of ECMO in pregnancy is safe and practica ble when performed in an experienced center and is generally associated with satisfactory results (41).

## Discussion

The physiological hypercoagulable state of pregnancy that is vital for maintaining the pregnancy, aiding the development of the fetus and controlling the blood loss post-delivery unfortunately increases the risk of thrombotic events. This increased risk is present throughout pregnancy and post-partum period. A high index of suspicion is important as the presenting signs and symptoms of PE are generally nonspecific which makes it challenging to distinguish the diagnosis from other life-threatening emergencies. Once diagnosed, prompt treatment will improve the chances of successful treatment. Thrombolysis remains the most-used and reasonably successful modality of treatment in pregnancy but should be avoided in the postpartum period as it can cause life-threatening haemorrhage. During the post-partum period, thrombectomy is the treatment of choice.

Once the acute treatment is taken care of, it is important to plan for intermediate and long-term anticoagulation. If still pregnant, then the mode of delivery and the timing of the anticoagulants during the peripartum period should be carefully worked out considering other obstetric factors. The women should be made aware of long term—possibly lifelong implications (such as avoiding combined oral contraceptive pills, need for anticoagulants in the future) of the PE.

All clinicians and pregnant women should be educated on the risk of VTE. Every woman needs to be risk assessed for VTE at the beginning of the pregnancy. They should be educated to remain vigilant for symptoms and report promptly. The clinicians should evaluate suspected VTE, and start appropriate therapy in a timely manner.

The need of the hour is to establish a validated diagnostic algorithm specific to the pregnant and postpartum women. There has been an attempt in this direction as discussed above. There is a need for a multidisciplinary guideline in management of this emergency. Thrombolysis remains the treatment of choice in the antenatal period and embolectomy is considered reasonable alternative in the post-partum period (6). There are reports of using combination of the treatments to improve successful treatment (23). After the successful treatment in pregnant women, a comprehensive care plan to manage the rest of pregnancy including delivery and post-partum period is vital. Current evidence points towards better survival rates in pregnancy and post-partum period, but as there are no randomised control trials, a publication bias cannot be ruled out. A randomised control study would help eliminating the possible bias affecting the results of the current studies.
